# Influence of clonidine induced sympathicolysis on anaemia tolerance in anaesthetized pigs

**DOI:** 10.1186/s12871-016-0258-2

**Published:** 2016-10-12

**Authors:** Patrick Lauscher, Harry Kertscho, Malte Krömker, Barbara Haberichter, Kai Zacharowski, Peter Rosenberger, Jens Meier

**Affiliations:** 1 Clinic of Anesthesiology and Intensive Care Medicine, Sana Klinikum Offenbach, Offenbach, Germany; 2 Division of Anaesthesiology, Balgrist University Hospital Zurich, Zurich, Switzerland; 3 Division of Kardiology, Department of Internal Medicine, University Hospital Frankfurt, Frankfurt am Main, Germany; 4 Department of Anesthesiology, Intensive Care Medicine and Pain Therapy, University Hospital Frankfurt, Frankfurt am Main, Germany; 5 Department of Anaesthesiology and Intensive Care Medicine, University Hospital Tübingen, Tübingen, Germany; 6 Department of Anesthesia and Intensive Care, Faculty of Medicine of the Kepler University Linz, 4021 Linz, Austria

**Keywords:** Anaemia, Hemodilution, Critical haematocrit, Oxygen consumption, Clonidine, Sympathicolysis

## Abstract

**Background:**

Clonidine effectively decreases perioperative mortality by reducing sympathetic tone. However, application of clonidine might also restrict anaemia tolerance due to impairment of compensatory mechanisms. Therefore, the influence of clonidine induced, short-term sympathicolysis on anaemia tolerance was assessed in anaesthetized pigs. We measured the effect of clonidine on anaemia tolerance and of the potential for macrohemodynamic alterations to constrain the acute anaemia compensatory mechanisms.

**Methods:**

After governmental approval, 14 anaesthetized pigs of either gender (Deutsche Landrasse, weight (mean ± SD) 24.1 ± 2.4 kg) were randomly assigned to intravenous saline or clonidine treatment (bolus: 20 μg · kg^−1^, continuous infusion: 15 μg · kg^−1^ · h^−1^). Thereafter, the animals were hemodiluted by exchange of whole blood for 6 % hydroxyethyl starch (MW 130.000/0.4) until the individual critical haemoglobin concentration (Hb_crit_) was reached. Primary outcome parameters were Hb_crit_ and the exchangeable blood volume (EBV) until Hb_crit_ was reached.

**Results:**

Hb_crit_ did not differ between both groups (values are median [interquartile range]: saline: 2.2 (2.0–2.5) g · dL^−1^ vs. clonidine: 2.1 (2.1–2.4) g · dL^−1^; n.s.). Furthermore, there was no difference in exchangeable blood volume (EBV) between both groups (saline: 88 (76–106) mL · kg^−1^ vs. clonidine: 92 (85–95) mL · kg^−1^; n.s.).

**Conclusion:**

Anaemia tolerance was not affected by clonidine induced sympathicolysis. Consequently, perioperative clonidine administration probably has not to be omitted in view of acute anaemia.

## Background

Allogeneic blood transfusions are expensive and, though safer than ever before, are still associated with potential infectious, immunologic, and immunosuppressive risks.

Therefore one pillar of modern patient blood management concepts is to decrease the number of perioperative blood transfusions by accepting low intraoperative haemoglobin concentrations without endangering tissue oxygenation. However, this decline of oxygen carrying capacity has to be compensated for by an increase of cardiac output, organ perfusion, and oxygen extraction ratio [1–3]. As long as these compensatory mechanisms can be utilized, remarkable degrees of anaemia can be survived, and less severe anaemia can be sustained without significant sequelae [4].

Presently, a considerable number of patients are treated with perioperative α_2_-agonists, either for sympathicolysis to reduce perioperative cardiac risk or for sedation during ICU hospitalization [5]. Perioperative α_2_-adrenergic agonists decrease global oxygen consumption [6] and improve perioperative myocardial morbidity and mortality [7, 8] by increasing coronary perfusion especially in ischemic heart disease [9], by improving coronary reserve, and by decreasing oxygen consumption [6, 10]. Furthermore, they are widely used for postoperative sedation in the ICU [11].

However, the influence of clonidine induced cardiovascular alterations on acute anaemia tolerance is currently unknown. Therefore we determined the effect of clonidine on anaemia tolerance and haemodynamic compensatory mechanisms of acute anaemia in a pig model of acute normovolemic hemodilution.

## Methods

The experimental protocol was approved by the Animal Care Committee (Regierungspräsidium Darmstadt, Hessen, F 143/18). The experiments were performed in 14 pigs (Deutsche Landrasse) of either sex (mean body weight 24.1 ± 2.4 kg). The animals were treated in accordance with the Principles of Laboratory Animal Care (National Institute of Health publications 86–23, 1985).

### Anaesthesia

Food was withheld one night prior to anaesthesia, but animals had free access to water. Midazolam (1.5 mg · kg^−1^) and ketamine (10 mg · kg^−1^) were administered intramuscularly prior to anaesthesia. Anaesthesia was induced with intravenous fentanyl (0.01 mg · kg^−1^), propofol (2 mg · kg^−1^), and vecuronium bromide (0.3 mg · kg^−1^) and maintained by continuous intravenous fentanyl (0.045 mg · kg^−1^ · h^−1^), propofol (10 mg · kg^−1^ · h^−1^), and midazolam (0.6 mg · kg^−1^ · h^−1^) infusion. Vecuronium bromide (1.0 mg · kg^−1^ · h^−1^) was administered continuously to maintain muscular relaxation and minimize muscular oxygen consumption. Estimated insensitive fluid losses were compensated for by intravenous administration of isotonic electrolyte solution (3 mL^−1^ · kg^−1^ · h^−1^; Sterofundin® ISO, B. Braun Melsungen AG, Melsungen, Germany).

A warming blanket (Warm Touch, Mallinckrodt/Convidien; Boulder, CO, USA) was used to maintain constant body temperature (baseline temperature ± 0.25 °C). Animals were intubated endotracheally and mechanically ventilated (FiO_2_ 0.21) at a rate of 12–15 min^−1^ and a positive end-expiratory pressure of 5 cmH_2_O. The minute volume was adjusted to maintain normocapnia, which was confirmed by multiple blood gas analyses.

### Instrumentation and monitoring

All animals were placed in supine position. A 12-lead electrocardiogram was recorded continuously during the entire experiment. Several intravascular catheters were placed using Seldinger’s technique to avoid stress related to surgical preparation. A Picco thermodilution catheter (PICCO Pulsiocath, PULSION Medical Systems AG, Munich, Germany) was placed in the right femoral artery to measure arterial pressure and continuously measure cardiac output.

A 20-gauge catheter was placed in the left femoral artery for phlebotomy, and a 16-gauge catheter inserted into a femoral vein to administer hydroxyethyl starch during hemodilution. A 16-gauge catheter was inserted through the left external jugular vein into the upper vena cava to administer anaesthetic agents and monitor central venous pressure; a pulmonary arterial catheter (7.5 Fr, Edwards Swan-Ganz, Baxter Healthcare, Irvine, CA) was inserted to sample mixed venous blood and monitor pulmonary arterial pressure. The catheter positions were verified by blood gas analysis and radiography.

### Experimental protocol

After instrumentation, a 60-min stabilization period was allowed for VO_2_ stabilization (see: Determination of Hbcrit) before the first set of data was collected (baseline, BL1). Blood volume was determined by indocyanine green dilution kinetics using the “whole blood method”[12]. The animals were then randomized into two experimental groups comprising seven animals each.

After randomization animals received either 20 μg · kg^−1^ clonidine over a 10-min period (clonidine group) or an equal volume of saline (control group) according to randomization. Thereafter, 15 μg · kg^−1^ · h^−1^ clonidine or the equal amount of saline was administered intravenously. After the initial bolus, a 60-min stabilization period elapsed, and a second set of data set was collected (BL2).

After baseline 2 (BL2) all animals were hemodiluted isovolemically with HES 6 % (MW 130.000/0.4) at an exchange rate of 1 mL · kg^−1^ · min^−1^ with a infuse/withdrawal dual syringe pump (Harvard Apparatus, Holliston, MA, US) until the critical haemoglobin concentration (Hb_crit_) was reached. The hemodilution procedure was performed in steps of 200 ml, after which a short break for hemodynamic measurements took place. This procedure was repeated until the critical haemoglobin concentration (time point Hb_crit_) was detected. Hb_crit_ was defined as a significant decrease in total body VO_2_ compared to the baseline value (see: Determination of Hbcrit). The total blood volume that was exchanged to reach Hb_crit_ was designated as the exchangeable blood volume (EBV).

### Measurements

Arterial and mixed venous PO_2_ and PCO_2_, pH, electrolytes, haematocrit and arterial serum lactate concentration was measured using a blood analysing system (Premier GEM 3000, Instrumentation Laboratory, Lexington, MA, USA). Haemoglobin concentration (Hb) and arterial haemoglobin-oxygen saturation (SaO_2_) were measured by spectrophotometry adjusted to swine haemoglobin (682 CO-Oximeter, Instrumentation Laboratory, Lexington, MA, USA). Oxygen transport and uptake were calculated as described in the Appendix.

### Data analysis

Six experimental data sets were used for calculations, defined as the baseline measurement (BL1), after clonidine medication (second baseline, after medication, BL2) and when 25, 50, 75, and 100 % of exchangeable blood volume (EBV) until Hb_crit_ were exchanged. These parameters are summarized in Table 2.

The main outcome parameter of the study was the critical haemoglobin concentration (Hb_crit_) and the exchangeable blood volume (EBV) until Hb_crit_ was reached. Secondary outcome parameters were several macrohemodynamic, oxygen transport, and tissue oxygenation parameters.

### Determination of Hb_crit_

**Fig. 1:**
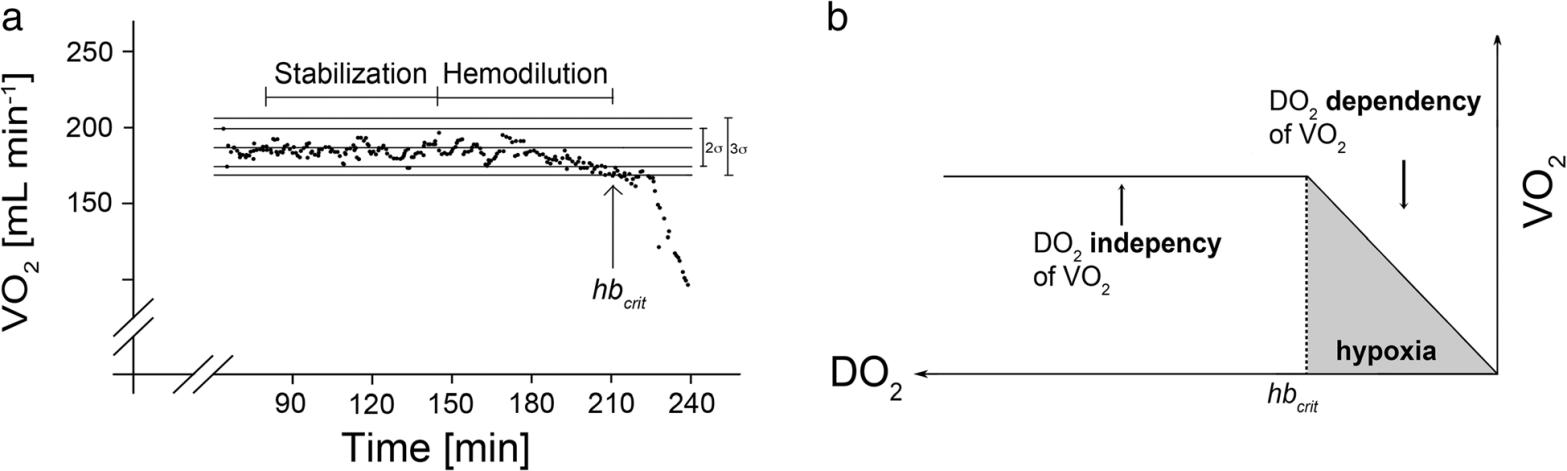
**a** Typical example of an oxygen consumption (VO_2_) recording in the course of the experimental protocol. A linear regression analysis including the calculation of standard deviation was performed with VO_2_ values collected during the 60-min stabilization period. During the subsequent hemodilution protocol, a critical limitation of DO_2_ was assumed, when the actually measured VO_2_ value decreased below the lower 3 s. **b** Dependency of oxygen consumption (VO_2_) on oxygen delivery (DO_2_) during normovolemic anemia. The graph presentation of Fig. 1 panel is reflected around a vertical axis (with the x-axis to the right of the y-axis) to match the decreasing VO_2_ along the timeline of panel a. In panel b DO_2_ decreases from left to right along the x-axis and VO_2_ decreases from top to bottom along the y-axis. Despite an initial decrease of DO_2_, VO_2_ remains stable over a long period (DO_2_ is independent of VO_2_). If a critical hemoglobin concentration (Hb_crit_) is reached, VO_2_ starts to decrease because of a critical restriction (DO_2_ dependent on VO_2_)

In steady state, tissue oxygen consumption (VO_2_) equals oxygen demand. However, when DO_2_ decreases (e.g. by hemodilution) below a critical value, VO_2_ becomes oxygen supply-dependent and decreases (Table 1, Fig. 1a). This sudden VO_2_ decrease reflects the onset of global tissue hypoxia [13], and the corresponding haemoglobin concentration is called the “critical” Hb concentration (Hb_crit_). Previous studies found that 100 % of animals died within 3 h after achieving Hb_crit_ [14].

**Table 1:** Macrohemodynamics

	Group	BL1	BL2	25 %	50 %	75 %	Hb_crit_
HR	*Saline*	*88 (87–107)*	84 (83–107)**	91 (84–109)**	*107 (85–113)*	*128 (95–132)*	130 (98–142)**
bpm	Clonidine	88 (79–114)	57 (53–62)**	64 (63–76)**	83 (79–88)	96 (91–116)	95 (94–101)**
MAP	*Saline*	*88 (69–107)*	87 (70–105)**	*87 (69–99)*	*87 (70–96)*	*80 (64–93)*	*53 (48–59)*
mmHg	Clonidine	85 (75–90)	113 (104–124)**	91 (83–99)	85 (79–88)	67 (66–79)	46 (27–52)
CI	*Saline*	*5,9 (5,4-6,3)*	6,0 (5,6-6,3)**	6,1 (5,4-6,9)**	6,9 (6,5-8,1)**	*7,3 (7,0-8,5)*	*7,9 (7,4-8,5)*
L min^−1^m^−2^	Clonidine	5,3 (4,6-5,6)	2,4 (2,2-3,4)**	4,0 (3,5-4,6)**	5,1 (4,3-5,7)**	6,5 (5,7-7,7)	6,9 (6,0-8,5)
SVRI	*Saline*	*2216 (1755–2404)*	2150 (1656–2391)**	1791 (1509–2072)**	*1500 (1321–1739)*	*1526 (989–1547)*	*677 (624–830)*
dyn sec cm^−5^m^−2^	Clonidine	2646 (1997–3196)	4812 (4649–5471)**	2678 (2077–3203)**	1980 (1736–2102)	1247 (1171–1492)	428 (354–559)
PVRI	*Saline*	*281 (276–332)*	*317 (284–361)*	*224 (205–308)*	*209 (194–221)*	*205 (190–264)*	*162 (135–173)*
dyn sec cm^−5^m^−2^	Clonidine	316 (262–389)	379 (327–457)	271 (248–360)	278 (262–285)	231 (225–248)	139 (123–177)
CPP	*Saline*	*62 (43–71)*	60 (46–70)**	*51 (41–63)*	*41 (37–61)*	*32 (32–58)*	*23 (13–27)*
mmHg	Clonidine	49 (43–62)	77 (71–84)**	53 (47–61)	46 (37–53)	35 (25–40)	6 (2–16)
LVPsys	*Saline*	121 (85–127)	122 (86–126)**	109 (87–121)**	102 (85–115)	95 (82–113)	77 (74–80)
mmHg	Clonidine	105 (101–113)	143 (138–157)**	121 (117–130)**	114 (106–119)	100 (90–108)	70 (52–89)
LVPedp	*Saline*	*12 (12–15)*	13 (12–15)**	15 (13–19)**	16 (16–19)**	*16 (15–17)*	*17 (16–23)*
mmHg	Clonidine	11 (9–19)	16 (15–29)**	17 (16–26)**	19 (18–26)**	14 (12–22)	22 (18–26)
MPAP	*Saline*	*22 (21–27)*	*23 (22–32)*	*24 (22–31)*	*25 (22–31)*	*26 (22–32)*	*26 (24–34)*
mmHg	Clonidine	25 (23–27)	26 (24–27)	25 (24–29)	27 (25–32)	30 (25–31)	27 (25–29)
LVPdtp_max_	*Saline*	*2210 (1705–2555)*	*2160 (1750–2528)*	*2400 (1950–3220)*	*2830 (2510–3630)*	*3630 (2748–3925)*	*2080 (1810–2805)*
mmHg s^−1^	Clonidine	2510 (2105–2935)	1644 (1525–2550)	2350 (2070–2755)	2450 (2179–3260)	2430 (2275–3105)	1710 (885–2145)
LVPdtp_min_	*Saline*	*−3070 (−5130--2666)*	*−3120 (−5015--2445)*	*−3290 (−4420--2465)*	*−3200 (−4680--2554)*	*−2850 (−4950--2680)*	−1820 (−2840--1195)**
mmHg^−1^	Clonidine	−2510 (−3530--2300)	−3800 (−3890--3235)	−3340 (−4065--2735)	−3080 (−3650--2900)	−2900 (−2950--2170)	−1100 (−1660--785)**
LVSWI	*Saline*	*642 (595–820)*	*688 (604–825)*	*686 (595–790)*	*752 (671–896)*	*686 (642–749)*	*631 (393–668)*
Nm 10^−3^m^−2^	Clonidine	435 (347–657)	610 (516–962)	717 (590–897)	664 (602–819)	609 (504–751)	409 (258–616)
RVSWI	*Saline*	*155 (148–216)*	*210 (172–231)*	*203 (178–238)*	*253 (190–286)*	*241 (178–272)*	*284 (231–299)*
Nm 10^−3^m^−2^	Clonidine	125 (116–200)	148 (119–229)	233 (169–264)	209 (179–282)	203 (186–292)	271 (229–346)

Parameters of macrohemodynamics. All values are presented as median and quartiles (Q_1_-Q_3_) for the investigated time points BL1 (baseline, premedication), BL2 (second baseline, after medication), 25 % (exchange of 25 % of exchangeable blood volume (EBV)), 50 % (exchange of 50 % of EBV), 75 % (exchange of 75 % of EBV), Hb_crit_ (critical haemoglobin concentration). **: *p* < 0.05 Saline vs. Clonidine

*HR* heart rate, *MAP* mean arterial pressure, *CI* cardiac output indexed to BSA, *SVRI* systemic vascular resistance indexed to BSA, *PVRI* pulmonary vascular resistance indexed to BSA, *CPP* coronary perfusion pressure, *LVPsys* systolic left ventricular pressure, *LVPedp* enddiastolic left ventricular pressure, *MPAP* mean pulmonary arterial pressure, *LVPdtp*_*max*_ maximum left ventricular pressure increase, *LVPdtp*_*min*_ maximum left ventricular pressure decrease, *LVSWI* left ventricular stroke work index, *RVSWI* right ventricular stroke work index

Total body VO_2_ was measured non-invasively at 1-min intervals using a DeltaTrac metabolic monitor (DeltaTrac IITM MBM-200, Datex, Helsinki, Finland) connected to the ventilator. Hb_crit_ was automatically detected using computer software especially designed for this purpose (DeltaCrit System) [15]. VO_2_ values collected during the 60-min stabilization period were included in an online regression analysis. The VO_2_ values measured during hemodilution were compared to the mean value predicted by the DeltaCrit system; if the measured value was outside a predefined range (3 × SD of the regression line), the VO_2_ was considered significantly decreased, and the computer alerted visually and acoustically. This time point was designated as Hb_crit_ (Fig. 1b).

### Statistical analysis

Prior to the study, a sample size analysis was conducted using a software package (PS, Power and Sample Size Calculation, Version 3.0, 2009, Vanderbilt University, Tennessee, USA) to estimate the appropriate number of animals. From former studies we anticipated the Hb_crit_ around a haemoglobin concentration of 2,7 g/dL (±0.55 g/dL). We determined a difference of 0.7 g/dL as clinical significant. With a two-sided α of 0.05 and a power of 80 %, seven animals per group were required for the Student´s *t*-test to detect differences in the critical haemoglobin concentration.

Between the saline and clonidine groups Hb_crit_ and EBV were normally distributed, therefore differences between the two study groups were tested using a student´s *t*-test for independent samples. Distributions of macrohemodynamic and oxygen transport data were tested by the Kolmogorov-Smirnov test. Not all data were normally distributed; therefore, data are presented as the median and quartiles (Q1–Q3). A repeated-measurement ANOVA on ranks was performed to detect differences between groups at the main time points: BL, AM, and 25, 50, 75 %, and Hb_crit_.

Post hoc analysis was performed with a Student-Newman-Keuls (SNK) test to account for multiple comparisons (Statistica 5.1, StatSoft, Tulsa, OK). Statistical significance was designated at *p* < 0.05 for all tests.

## Results

No differences were detected at BL between the study groups in age, sex, weight, and all other variables. Values are median (Q_1_–Q_3_)

### Primary end points: Hb_crit_ and EBV

**Fig. 2:**
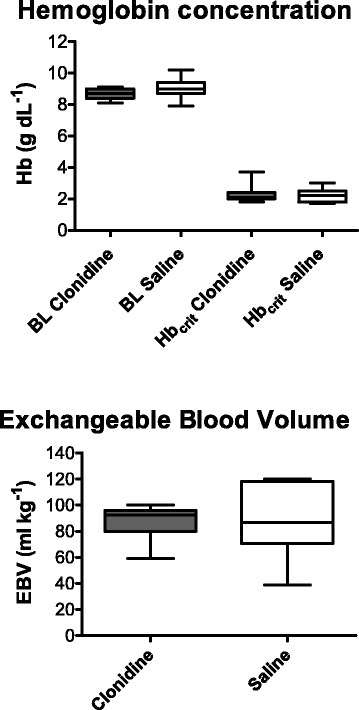
Haemoglobin concentration at BL and Hb_crit._ Grouped boxplots of the arterial haemoglobin concentration (g · dL^−1^) at BL and Hb_crit_. The results are presented pairwise as boxplots (median line) with whiskers (min to max). Data from the clonidine group are coloured white, and saline group data are dark grey. No difference was demonstrated between the groups. BL (baseline, premedication); Hb_crit_ (critical haemoglobin concentration). Exchangeable blood volume (EBV). Boxplots of the exchangeable blood volume (mL · kg^−1^) until Hb_crit_ was reached. The results are presented pairwise as the median and quartiles (Q_1_-Q_3_). No differences were demonstrated between the groups. Hb_crit_ (critical haemoglobin concentration)

The *critical* haemoglobin concentration (Hb_crit_) was reached at 2.2 (2.0–2.5) g · dL^−1^ in the saline group and 2.1 (2.1–2.4) g · dL^−1^ in the clonidine group (Fig. 2; n.s.). Hemodilution to Hb_crit_ required a hydroxyethyl starch exchange of 88 (76–106) mL · kg^−1^ blood in the saline group and 92 (85–95) mL · kg^−1^ in the clonidine group, lasting approximately 83 min (n.s.). This corresponded to 115 % (86–166 %) of the blood volume at BL in the saline group and 121 % (108–124 %) in the clonidine group (Fig. 2; n.s.).

### Secondary end points: haemodynamics and myocardial function

**Fig. 3:**
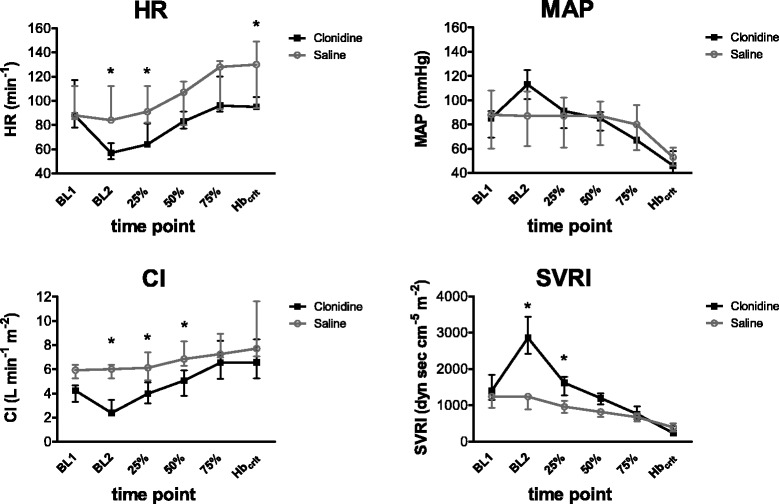
Cardiovascular parameters in anaesthetized pigs: heart rate (HR); mean arterial pressure (MAP); cardiac index (CI); systemic vascular resistance indexed to body surface area (SVRI) for the saline group (*light grey*) and the clonidine group (*black*) during baseline (BL1), medication (BL2), and the percentile hemodilution steps 25, 50, and 75 % until Hb_crit_ (critical haemoglobin concentration). Median and quartiles (Q_1_-Q_3_). *p < 0.05

Haemodynamic data are summarized in Table 1 and in Fig. 3. During haemodilution to Hb_crit_, cardiac index (CI) was significantly lower at BL2, 25 %, and 50 % time points in the clonidine group. Heart rate (HR) was also significantly lower at BL2, 25 %, and Hb_crit_ in the clonidine group. Mean arterial pressure (MAP) and coronary perfusion pressure (CPP) showed no intergroup differences, despite the significantly higher values in the clonidine group at BL2. Systemic vascular resistance (SVRI), left ventricular systolic (LVP_sys_), and left ventricular end diastolic pressure (LVP_edp_) were significantly higher in the clonidine group at BL2 and at 25 %, due to clonidine’s initial α_1_–mediated vasoconstriction. The significantly higher SVRI, LVP_sys_, and LVP_edp_ were not maintained during the progression to Hb_crit_.

Pulmonary vascular resistance (PVRI) and mean pulmonary pressure (MPAP) similarly trended in both groups and showed no statistically significant change. The left ventricular stroke work index (LVSWI) and right ventricular stroke work index (RVSWI) were similar between the two groups during the procedure. Left ventricular contractility (LVP_dtpmax_) showed no intergroup difference, but the left ventricular relaxation (LVP_dtpmin_) was significantly lower in the clonidine group at Hb_crit_.

### Secondary end points: oxygen transport and tissue oxygenation

**Fig. 4:**
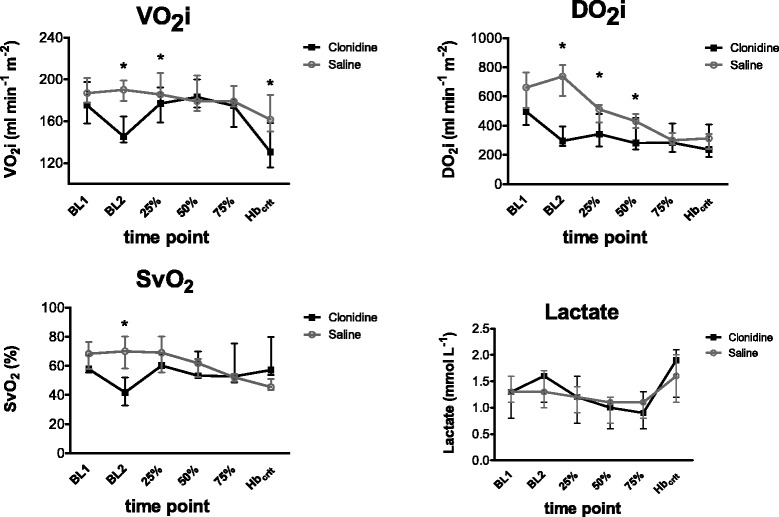
Oxygen transport and tissue oxygenation in anaesthetized pigs: measured oxygen consumption (VO_2_mi), oxygen supply (DO_2_i), mixed-venous oxygen saturation of haemoglobin (SvO_2_), and arterial serum lactate for the saline group (*light grey*) and the clonidine group (*black*) during baseline (BL), medication (BL2), and the percentile hemodilution steps 25, 50, and 75 % until Hb_crit_ (critical haemoglobin concentration). Median and quartiles (Q_1_-Q_3_). * *p* < 0.05

Oxygen transport and tissue oxygenation variables are presented in Table 2 and in Fig. 4. Changes in oxygen transport parameters and tissue oxygenation at BL2 and during hemodilution to Hb_crit_ were similar between the groups.

**Table 2:** Oxygen transport and tissue oxygenation

	Group	BL1	BL2	25 %	50 %	75 %	Hb_crit_
Hb	*Saline*	*9,0 (8,9*–*9,3)*	*8,6 (8,5*–*9,2)*	*6,0 (5,6*–6,4)**	*4,3 (3,9*–*4,5)*	*3,0 (2,7*–*3,1)*	*2,2 (2–2,5)*
g dl^−1^	Clonidine	8,7 (8,5–9)	8,7 (8,4–8,9)	6,5 (6,1–6,7)**	4,2 (4,1–4,7)	3,1 (2,9–3,4)	2,1 (2,1–2,4)
paO_2_	*Saline*	*90 (89–102)*	*89 (88–91)*	*93 (84–95)*	*93 (92–97)*	*94 (88–98)*	*108 (93–113)*
mmHg	Clonidine	92 (85–101)	94 (90–109)	85 (81–101)	93 (80–104)	93 (81–102)	109 (94–122)
pvO_2_	*Saline*	*36 (36–38)*	37 (36–44)**	*41 (36–44)*	*35 (32–37)*	*36 (33–42)*	*26 (22–28)*
mmHg	Clonidine	43 (41–45)	27 (26–32)**	32 (31–36)	36 (33–40)	29 (28–31)	26 (24–32)
CaO_2_	*Saline*	*12,2 (12–12,5)*	*11,6 (11,5*–*12,3)*	*8,3 (7,7*–8,6)**	*6,1 (5,4*–*6,2)*	*4,2 (3,8*–*4,5)*	*3,3 (3–3,6)*
ml dl^−1^	Clonidine	11,8 (11,5–12,2)	11,8 (11,5–11,9)	8,9 (8,3–9,1)**	5,9 (5,7–6,4)	4,4 (4,1–4,8)	3,2 (3,1–3,4)
avDO_2_	*Saline*	*3,9 (3,2*–*4,8)*	*3,4 (3,1*–4,6)**	*2,4 (1,8*–*3,2)*	*2,3 (1,9*–*2,8)*	*2,0 (1,9*–*2,2)*	*1,9 (1,7*–*2,1)*
ml dl^−1^	Clonidine	4,7 (4,2–5,0)	6,3 (5,6–7,5)**	3,4 (2,5–4,0)	2,8 (2,3–2,8)	2,1 (1,4–2,1)	1,4 (1,1–1,9)
O_2_-ER	*Saline*	*31,3 (25,3*–*39,3)*	*29,7 (26,2*–38,3)**	*31,8 (23,6*–*41,5)*	*40,3 (37–46,4)*	*49,3 (48,1*–*49,9)*	*56,7 (54,1*–*58,2)*
%	Clonidine	41,2 (34,8–43,7)	58,5 (47,5–63,4)**	41,3 (31,3–44,2)	47,5 (37,4–48,2)	49,0 (28,5–50,7)	44,7 (34,2–49,1)
SaO_2_	*Saline*	98,7 (97,9–99,1)	98,5 (98,4–98,7)	98,7 (97,9–99,0)	99,0 (98,7–99,3)	99,1 (98,7–99,2)	99,3 (99,0–99,5)
%	Clonidine	98,6 (98,1–99,0)	98,6 (98,0–99,1)	98,3 (98,3–98,7)	98,6 (98,0–98,9)	98,9 (98,2–99,0)	99,1 (98,9–99,1)
SvO_2_	*Saline*	*68,4 (59,4*–*75,5)*	*70,0 (61,8*–73,5)**	*69,1 (59–76,6)*	*61,9 (55,4*–*64,4)*	*52,2 (51,5*–*53,4)*	*45,5 (44,3*–*48,3)*
%	Clonidine	57,8 (56,1–65,0)	41,7 (35,8–51,3)**	60,2 (55,7–68,9)	53,2 (53,2–63,5)	52,8 (50,3–71,0)	57,1 (55,5–70,0)
DO_2i_	*Saline*	*607 (569–623)*	636 (612–677)**	512 (455–542)**	430 (393–469)**	*300 (284–348)*	*313 (266–336)*
ml min^−1^ m^−2^	Clonidine	512 (503–565)	269 (261–387)**	342 (288–418)**	281 (258–382)**	283 (228–394)	236 (190–341)
VO_2im_	*Saline*	187 (180–196)	190 (183–197)**	185 (183–197)**	179 (170–196)	179 (175–193)	162 (153–181)**
ml min^−1^m^−2^	Clonidine	175 (161–193)	145 (140–159)**	181 (168–187)**	183 (174–199)	175 (161–179)	130 (122–157)**
VO_2ic_	*Saline*	*209 (188–247)*	*231 (131–259)*	*152 (118–211)*	*162 (144–198)*	*161 (147–178)*	169 (157–201)**
ml min^−1^m^−2^	Clonidine	205 (142–223)	171 (129–236)	146 (102–157)	132 (88–146)	123 (94–166)	91 (53–158)**
Lactate	*Saline*	*1,3 (1,2*–*1,5)*	*1,3 (1,1*–*1,6)*	*1,2 (1–1,4)*	*1,1 (0,9*–*1,2)*	*1,1 (1–1,1)*	*1,6 (1,3*–*1,9)*
mmol l^−1^	Clonidine	1,3 (1–1,5)	1,6 (1,2–1,6)	1,2 (0,9–1,4)	1,0 (0,8–1,2)	0,9 (0,8–1,2)	1,9 (1,4–2,1)

Parameters of oxygen transport and tissue oxygenation. All values are presented as median and quartiles (Q_1_–Q_3_) for the investigated time points BL1 (baseline, premedication), BL2 (second baseline, after medication), 25 % (exchange of 25 % of EBV), 50 % (exchange of 50 % of EBV), 75 % (exchange of 75 % of EBV), Hb_crit_ (critical hemoglobin concentration). **: *p* < 0.05 Saline vs. Clonidine

*Hb* hemoglobin concentration, *PaO*_*2*_ arterial oxygen partial pressure, *PvO*_*2*_ central venous oxygen partial pressure, *CaO*_*2*_ arterial oxygen content, *avDO*_*2*_ arterio-venous difference in oxygen content, *O*_*2*_ *nER* oxygen extraction, *SaO*_*2*_ arterial hemoglobin saturation, *SvO*_*2*_ mixed venous hemoglobin saturation, *DO*_*2i*_ oxygen delivery indexed to BSA, *VO*_*2im*_ measured oxygen consumption indexed to BSA, *VO*_*2ic*_ calculated oxygen consumption, *Lactate* arterial serum lactate concentration, *pH* negative logarithm of the molar concentration of dissolved hydronium ions in a solution

Oxygen delivery (DO_2_) and oxygen consumption (VO_2_ mi) were significantly lower in the clonidine group at BL2, 25 %, and 50 %. Arterial serum lactate and arterial pH did not differ significantly between the groups and remained stable throughout the procedure.

At BL2, arterio-venous difference in (avDO_2_) and oxygen extraction ratio (O_2_–ER) were significantly higher, whereas mixed-venous oxygen saturation (SvO_2_) was significantly lower in the clonidine group. During hemodilution from baseline to the study endpoint (Hb_crit_), no ventricular arrhythmia, ectopia, ST-level changes, or other electrocardiographic arrhythmias were encountered in either group.

## Discussion

The present study indicates foremost that a high dose of intravenous clonidine did not critically restrict acute anaemia compensatory mechanisms in a swine model. This is reflected in identical critical haemoglobin concentrations and exchangeable blood volumes observed in both study groups.

Alpha-2 agonists mediate their cardioprotective effects by attenuated catecholamine release and thus partially inhibited stress-induced tachycardia. Apart from these haemodynamic effects, α_2_-agonists also induce analgesia, anxiolysis, and sedation through central presynaptic α_2_-adrenergic receptors [16]. Furthermore, whole body and myocardial oxygen consumption decrease under α_2_-agonist treatment [6].

Increased heart rate is one of the compensatory mechanisms for acute dilutional anaemia, and myocardial oxygen consumption is an important determinant of the outer limits of this compensatory mechanism; yet, the influence of α_2_-agonists on this effect and by that on anaemia tolerance is unclear. Potentially, clonidine induced reduction in heart rate may impair compensatory potential, but conversely, the decreased oxygen consumption may improve anaemia tolerance. To date, no existing study comprehensively investigates the effects of perioperative α_2_-agonists on anaemia tolerance.

Unfortunately, "anaemia tolerance" is poorly defined, and consequently, its limits are difficult to measure. For example, short periods of anaemia and concomitant tissue hypoxia can be sustained without any sequelae [4], although the individual anaemia tolerance of several organs may be exceeded. Furthermore, organ specific markers of tissue hypoxia (e. g. ECG changes, cerebral function, urinary output) likely indicate that the outer limit of anaemia has been surpassed at an earlier time point, but do not reflect "anaemia tolerance" comprehensively because the complete organism is not considered [17–19].

Several studies demonstrate that a significant decline in whole body oxygen consumption is accompanied by inadequate tissue oxygenation, and as a consequence this parameter has been used regularly as measure for anaemia tolerance [20, 21]. The corresponding haemoglobin concentration is called "critical haemoglobin concentration (Hb_crit_)." Several experimental studies have observed haemodynamic decompensation [22], increased lactate, and elevate catecholamine concentrations once Hb_crit_ was reached that resulted in the deaths of all animals within 3 h [14]. In a recent study, we showed that Hb_crit_ is accompanied by widespread tissue hypoxia in several organs, determined by quantification of hypoxia-specific changes in protein and RNA concentrations [20].

While this approach does not guarantee that the tissues are undamaged by hypoxia before Hb_crit_ is reached, it is apparent that upon reaching the Hb_crit_, the individual animals are similarly restricted in tissue oxygenation, therefore, Hb_crit_ is a reasonable measure of global anaemia tolerance [20].

Although no pig specific data on clonidine dosage exist, the dosage used in our model was chosen deliberately high compared to the rather few existing studies. While Iber et al. established sympathicolysis with 1/10 of our dose in a swine model [23], we failed to identify hemodynamic changes in several dose finding experiments with this approach. Finally, we titrated the medication up to a bolus of 20 μg · kg^−1^ and a continuous rate of 15 μg · kg^−1^ · h^−1^ to ensure a hemodynamic relevant sympatholytic effect of the drug tested. This is bolstered by the distinct increase in mean arterial pressure and a concomitant cardiac output decline following the initial clonidine bolus. The initial peripheral vasoconstriction is mediated by the partial affinity of clonidine to postsynaptic α_1_-receptors, until the higher affinity to central presynaptic α_2_-receptors prevails [24]. However, clonidine concentration was not measured, and as a consequence we cannot guarantee that clonidine concentration was consistent throughout the protocol.

The observed haemodynamic effects essentially depend on the chosen therapeutic scheme; in daily clinical practice, clonidine dosage is much lower, and the subsequent haemodynamic changes differ essentially from our observation. After an initial, modest increase, the mean arterial blood pressure and heart rate usually decline (Table 1, Fig. 3). Although our model does not mimic this clinical approach, we can deduce from our data that clonidine only slightly influences anaemia tolerance. Hb_crit_ has been observed at concentrations as low as 2.1 g · dL^−1^ during our relative clonidine overdosage; the haemodynamic changes in clonidine treated animals were comparable to those in untreated animals during hemodilution, therefore normal clonidine dosage will presumably only negligibly influence haemodynamic changes during acute anaemia.

One possible explanation for the mild hemodynamic effects observed during hemodilution might originate from the specific compensation mechanisms during acute anemia. In anesthetized subjects, the reduction of oxygen content of the blood is compensated by an increase in cardiac output via an increase in stroke volume [25, 26]. This increase in stroke volume and thus cardiac output is mainly effected by the Frank-Starling mechanism: the decrease of blood viscosity due to hemodilution reduces the peripheral resistance by shear stress induced NO-liberation from the vessel-endothelium and thus increases venous return [27, 28]. Therefore, in contrast to the pathophysiology of hypovolemic/haemorrhagic shock, in acute, normovolemic anemia oxygen delivery to the tissues is not maintained by increased sympathetic tone, but by the Frank-Starling mechanism [29]. This might explain the lack of hemodynamic changes by a drug that basically mediates its cardioprotective effects by attenuation of catecholamine release.

Bolus clonidine administration reduced VO_2_ in our model and may therefore have optimized the oxygen consumption/delivery balance. Several authors have similarly observed decreased VO_2_ and energy expenditure after clonidine administration. [6, 10, 30] However, this effect was limited in our model, occurring only at the initial time points following administration (BL2, 25 %) where it could be attributed to severe bradycardia. Presumably, haemodynamic changes during hemodilution were too small to influence VO_2_ significantly.

Underdosage of clonidine seems rather unlikely given the observed haemodynamic changes, therefore the mechanisms underlying these findings remain unknown. It is particularly unclear whether a more distinctive effect on VO_2_ may have caused even more pronounced anaemia tolerance.

Despite significant haemodynamic differences between the two experimental groups, only negligible differences were observed in oxygen transport and tissue oxygenation. Even at very low Hb levels, there were no differences observed in oxygen consumption, oxygen delivery, arterial serum lactate concentration, and acid base balance between both study groups. It can therefore be surmised that the high clonidine dose did not critically restrict tissue oxygenation during acute anaemia.

Several study limitations warrant discussion. We investigated the effects of clonidine on anaemia tolerance in young, healthy pigs. Pigs are a generally accepted animal model for hemodynamic shock and multiple studies have shown that the limits of oxygen supply are comparable for anesthetized, paralyzed healthy pigs and anesthetized, paralyzed, otherwise healthy humans [4, 14, 31–34]. However, in clinical practice, patients with concomitant cardiac risks or comorbidities are the primary population administered perioperative α_2_ -adrenergic agonists [7, 8]. Consequently, our model may differ from clinical practice because compensatory mechanisms for acute anaemia may be different between healthy patients and those with significant cardiac disease. However, our results help to elucidate underlying mechanisms of anemia tolerance during clonidine medication.

We demonstrated that Hb_crit_ did not differ between control and clonidine treated animals, but this does not indicate that moderate anaemia (Hb 8–10 g · dL^−1^) can be sustained similarly in both groups in clinical practice. Using our model we cannot rule out that in some organs oxygen transport and tissue oxygenation are restricted more severely by clonidine than others, and as a result, anaemia tolerance may differ between different organs.

Dexmedetomidine was described to have even stronger α_2_-selective effects than clonidine [35]. A comparison of both drugs during acute anemia might reveal further insight into the effects of α_2_-agonists during acute anemia. β-adrenergic receptor antagonists are a common medication in elderly patients and have comparable hemodynamic effects to α_2_-agonists. Perioperative ß-blockade might influence cardiac compensatory mechanisms for acute blood losses and thus reduce the patients natural anemia tolerance [36].

Finally, since this study was planned using sample size calculations based on the Hb_crit_ as the main outcome parameter, it might be underpowered to detect all differences in the secondary outcome parameters (hemodynamics, oxygen transport)- at each timepoint with multiple comparisons.

In summary, we demonstrated that the limitations of extreme anaemia are independent of clonidine therapy, and despite the study limitations, this indicates a similar anaemia tolerance in both experimental groups. Clinically, these results suggest that clonidine administration alone does not justify blood transfusion at higher haemoglobin concentrations. However, despite these results, transfusion at higher thresholds may still be necessary due to concomitant diseases that triggered clonidine therapy.

## Conclusion

In conclusion, extended central sympathicolysis with clonidine has no influence on the critical haemoglobin concentration and global anaemia tolerance in young, healthy pigs. Based on these findings, blood transfusion cannot be justified at considerably higher haemoglobin concentrations after administering clonidine alone. This approach supports identical transfusion schemata for patients with or without perioperative central sympathicolysis, however safety studies in humans, particularly cardiac compromised patients, are needed to verify our results clinically.

## Appendix

Body surface area (BSA in m^2^) was calculated according to Holt et al. [37]

$$ BSA=k\times B{W}^{\frac{2}{3}} $$

where BW = body weight (in kg) and k = 9.

Cardiac index was calculated as:

$$ CI=CO/BSA $$

where CO is cardiac output.

Peripheral vascular resistance index was calculated as:

$$ SVRI=\left( MAP-CVP\right)\times \alpha /CI $$

where SVRI is systemic vascular resistance, α is a constant factor necessary to adjust the formula to the different dimensions, CVP is central venous pressure.

Arterial O_2_ content (CaO_2_) was calculated as:

$$ Ca{O}_2=\left(Hb\times Sa{O}_2\times 1.34\right)+pa{O}_2\times 0.003 $$

Total body O_2_ delivery (DO_2_I) was calculated as follows:

$$ D{O}_2I=CI\times Ca{O}_2 $$
